# Sandwich Culture Platforms to Investigate the Roles of Stiffness Gradients and Cell–Matrix Adhesions in Cancer Cell Migration

**DOI:** 10.3390/cancers15061729

**Published:** 2023-03-12

**Authors:** Evangelia Bouzos, Prashanth Asuri

**Affiliations:** Department of Bioengineering, Santa Clara University, Santa Clara, CA 95053, USA

**Keywords:** cell migration, stiffness gradients, cell–matrix adhesions, adhesion-dependent migration, Rho GTPases, mechanotransductive signaling

## Abstract

**Simple Summary:**

Metastasis is the primary cause of death in cancer patients, yet there is a shortage of highly potent anti-metastatic drugs. Screening for anti-metastatic compounds involves studying cell migration by primarily using two-dimensional platforms, which makes migration along one plane possible. However, the spatial arrangement of biological signals in vivo is more complicated, especially when migration is guided by matrix stiffness gradients. In this work, we sought to develop a sandwich culture platform where cells are placed at the interface between a stiff tissue culture plate and soft alginate gel to study the role that transitions in stiffness have in cell migration. We specifically looked at the effects of matrix stiffness and mechanotransductive signaling on both adhesion-dependent and -independent modes of cell migration. The results reported could facilitate the development of optimal in vitro platforms to discover therapeutic strategies against tumor cell motility and invasion.

**Abstract:**

Given the key role of cell migration in cancer metastasis, there is a critical need for in vitro models that better capture the complexities of in vivo cancer cell microenvironments. Using both two-dimensional (2D) and three-dimensional (3D) culture models, recent research has demonstrated the role of both matrix and ligand densities in cell migration. Here, we leveraged our previously developed 2.5D sandwich culture platform to foster a greater understanding of the adhesion-dependent migration of glioblastoma cells with a stiffness gradient. Using this model, we demonstrated the differential role of stiffness gradients in migration in the presence and absence of adhesion moieties. Furthermore, we observed a positive correlation between the density of cell adhesion moieties and migration, and a diminished role of stiffness gradients at higher densities of adhesion moieties. These results, i.e., the reduced impact of stiffness gradients on adhesion-dependent migration relative to adhesion-independent migration, were confirmed using inhibitors of both mechanotransduction and cell adhesion. Taken together, our work demonstrates the utility of sandwich culture platforms that present stiffness gradients to study both adhesion-dependent and -independent cell migration and to help expand the existing portfolio of in vitro models of cancer metastasis.

## 1. Introduction

Cell migration is an essential, multistep, and integrated process that plays a key role in many biological phenomena, such as embryogenesis, wound healing, and disease progression [1,2,3,4,5,6]. In embryogenesis, the migration of cells makes the differentiation of cell type and function possible. In wound healing, the migration of leukocytes to the site of injury initiates the immune response, and the migration of fibroblasts and vascular endothelial cells begins the healing process. Cell migration is also an important aspect of disease progression in conditions such as arthritis and cancer metastasis [1,4,7,8,9]. Cancer cell migration and invasion into adjacent tissues is required for metastasis, but the molecular mechanisms of cancer metastasis are difficult to study due to the complexity of the numerous pathways involved [10,11,12,13]. For instance, it is known that the interactions of cell and extracellular matrix (ECM) stiffness, and cell binding domains are vital to cancer cell migration. Demoplasia and tumor stiffening are the result of a common fibrotic response to ECM accumulation within the tumor microenvironment [14,15,16]. Tumor stiffening due to increased ECM allows the tumor to grow and displace host tissue. Additionally, it encourages invasion into surrounding host tissues and allows cell–ECM interactions to increase. Cancer cells are connected to the ECM through integrins at cell–ECM adhesion sites, and cell binding to specific domains can cause a cascade of signaling pathways that enable the cell to respond to its environment and determine its invasive potential [16,17,18]. Therefore, developing in vitro platforms to better understand the relationships among matrix stiffness, adhesion binding, and cell migration could enable research on and identification of potential targets that inhibit cancer cell metastasis to be performed.

Previous work has demonstrated the impact of mechanical, chemical, and biological cues on cell migration [3,6,19,20,21]. Physical gradients have been developed to observe the effects that matrix stiffness and topography have on cell migration, and chemical gradients (based on both soluble and insoluble factors) have been used to model the effect of the type and density of functional molecules, such as ECM proteins, growth factors, and other signaling molecules. Combinations of mechanical and chemical gradients have also been used to more closely model the complex physiological environment that cells experience in vivo [3]. Most cell migration studies have exploited two-dimensional (2D) cell culture systems, but cancer cells experience mechanical and chemical signals in three dimensions in vivo. In order to improve upon the existing in vitro models of cell migration and study the role of transitions in stiffness in cell migration, recent research has developed three-dimensional (3D) culture platforms to better mimic the native cell microenvironment and study natural cell behavior, as some cells exhibit unnatural behavior when grown on 2D scaffolds [22,23,24,25,26]. For example, human breast epithelial cells in a 2D environment have been shown to develop similarly to tumors but exhibit normal development when cultured in 3D space [27]. Therefore, the development of platforms that closely mimic the natural 3D cell environment is essential to studying cancer cell migration. Recent studies have demonstrated that matrix stiffness and cell adhesion play important roles in 3D cell migration and that a balance of biophysical and biochemical signals governs migration in 3D environments [5,28,29].

Our own lab developed a sandwich culture platform wherein cells are placed at the interface between stiff tissue culture polystyrene (TCPS) and soft alginate, as a simple, yet relevant model to investigate the role of stiffness gradients in cell migration [30]. However, since unmodified alginate does not support cell adhesion, this platform was limited to the study of the role of adhesion-independent migration resulting from a stiffness gradient. Given the prevalence of adhesion-dependent migration in vivo [3,23], we were highly motivated to extend the utility of this platform to model and characterize adhesion-dependent cell migration with the inclusion of adhesion moieties, such as arginyl-glycyl-aspartic acid (RGD) or collagen. Specifically, we were curious to probe the following related questions: How does the inclusion of adhesion moieties affect cell migration? Do cells switch from the adhesion-independent mode to adhesion-dependent mode of migration? What are the relative roles of adhesion and stiffness in cell migration? Are these roles complementary or substitutive? Does the development of platforms that make the independent control of adhesion and stiffness possible expand on the current mechanistic underpinnings of cell migration? To address some of the aforementioned questions, we used an alginate-based 2.5D platform previously developed in our lab to independently study the role of mechanical and biochemical properties in cell migration. To probe the role of cell–matrix adhesions in migration, we used hydrogels prepared with alginate tethered with an RGD ligand or alginate blended with collagen. Using this model system, we examined the individual and possible synergistic effects of stiffness gradients and density of adhesion ligands on cell migration, as well as the role of signaling molecules in cell migration in response to biochemical and mechanical stimuli.

## 2. Materials and Methods

### 2.1. Materials

Human U-87 and U-251 glioblastoma cells were obtained from ATCC (Manassas, VA, USA); Dulbecco’s modified Eagle medium (DMEM), from Mediatech (Manassas, VA, USA); fetal bovine serum (FBS) and penicillin–streptomycin, from Invitrogen (Carlsbad, CA, USA); poly-L-lysine solution (0.01%), from Sigma Aldrich (Saint Louis, MO, USA); sodium pyruvate, MEM non-essential amino acids, and GlutaMax, from Life Technologies (Carlsbad, CA, USA); and trypsin, from CellGro (Manassas, VA, USA). A WST cell proliferation assay kit was purchased from Dojindo Molecular Technologies (Rockville, MD, USA). High-viscosity alginic acid sodium salt from brown algae (alginate) was purchased from Sigma Aldrich (Saint Louis, MO, USA); sterile alginate covalently coupled with GRGDSP, from FMC BioPolymer (Philadelphia, PA, USA); rat-tail collagen, from Advanced BioMatrix (San Diego, CA, USA); and agarose (low melting), from Thermo Fisher Scientific (Waltham, MA, USA), used as received. The chemical inhibitor of the RhoA-ROCK pathway (Y-27632) was purchased from Selleck Chemicals (Houston, TX, USA), and that of the Rac pathway (NSC23766), from EMD Biosciences (La Jolla, CA, USA). Integrin inhibitor RGD was purchased from Selleck Chemicals (Houston, TX, USA); GRGDSP, from Sigma Aldrich (Saint Louis, MO, USA); and cilengitide, from MedChem Express (Monmouth Junction, NJ, USA).

### 2.2. Preparation of Alginate Stocks for Migration Assays

Unmodified alginate samples were prepared by mixing high-viscosity alginic acid sodium salt from brown algae (Sigma Aldrich, St. Louis, MO, USA) in DI water to form 3% *w*/*v* stock solution. The mixture was allowed to homogenize under magnetic stirring for 30 min and was autoclaved at 121 °C for 20 min for sterilization. Sterile alginate covalently coupled with GRGDSP (hereafter referred to as RGD-modified alginate) was used as received to make 3% *w*/*v* stock solution. Collagen was obtained as sterile solution (4 mg/mL) and used as received.

### 2.3. Cell Culture

Standard mammalian cell culture methods were used to maintain and passage U87 and U251 glioblastoma cells. The cells were cultured on standard 100 mm cell culture plates (Greiner Bio-One, Monroe, CA, USA) to 60–80% confluency in a 37 °C incubator with 5% CO_2_ humidity. DMEM with 10% FBS, MEM non-essential amino acids, 1% penicillin–streptomycin, and sodium pyruvate was used to maintain the cells, and 0.25% trypsin was used to subculture the cells.

### 2.4. Migration Assay Setup

In the migration assays, we followed the protocol as previously described [30]. Briefly, ca. 12,000 cells per well were seeded into 48-well plates and allowed to grow for 48 h, after which the cell culture medium was replaced with 300 µL of alginate solution (either unmodified alginate, RGD-modified alginate, a mix of unmodified and RGD-modified alginate, or unmodified alginate containing collagen). A volume of 300 µL of 100 mM CaCl_2_ solution was added to each well containing cells and alginate solution for ca. 5 min to initiate gelation, after which CaCl_2_ was replaced with 300 µL of fresh medium. The cell culture medium was replaced every 48 h until the end of the experiment. In experiments involving agarose-coated substrates, the well plates were first coated with agarose (by adding agarose solutions to the well plates to form ca. 0.1 mm thick films and letting the solutions harden at room temperature), followed by coating with poly-L-lysine solution for one hour to achieve cell attachment. In experiments involving the inhibition of pathways of interest (Rho GTPase signaling or integrin binding), commercially available inhibitors were added to the cell culture medium on day 0, i.e., immediately after alginate gelation. The concentrations of inhibitors added were based on the previous literature; Rho GTPase inhibitor concentrations were 20 µM Y-27632 and 100 µM NSC23766 [31,32], and integrin inhibitors concentrations were 200 µM RGD, 200 µM GRGDSP, and 5 µM cilengitide [33,34,35].

### 2.5. Analysis of Cell Migration

After three days, the medium was replaced with an equivalent volume of 50 mM EDTA (purchased from BioRad, Hercules, CA, USA) and incubated for 30 min at 37 °C and 5% CO_2_. The digested alginate from each well was individually centrifuged for 2 min at 1500 rpm, and the cells obtained were quantified using the commercially available, formazan-based WST assay as per the manufacturer’s instructions. Briefly, the cells were resuspended in WST solution diluted in medium (1:5 *v*/*v*). The WST–medium mix was also added to the well plates post-alginate digestion to quantify the cells that did not migrate into alginate. These WST solutions were then incubated for 4 h at 37 °C, followed by centrifugation to remove the cells. Absorbance was then measured at 570 nm using Tecan Infinite 200 PRO (Durham, NC, USA). The WST absorbance reading of the alginate digest was divided by the sum of WST readings of the digest and well plate to calculate the percent migration for each well. Qualitative microscopic analyses were also performed on the sandwich cultures prior to alginate digestion to track and observe the cells that migrated into alginate.

## 3. Results

### 3.1. Alginate Concentration Affects 2.5D Cell Migration

**Figure 1:**
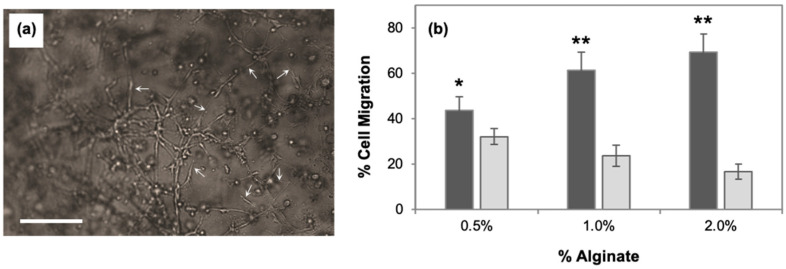
(**a**) Representative phase-contrast image showing migrated U87 cells into RGD-modified alginate scaffolds on day 3. The scale bar depicts 100 µm. The image shows that cells extended processes in RGD-modified alginate (as indicated by the white arrows). (**b**) Migration of U87 cells into 0.5%, 1%, and 2% RGD-modified (dark grey) and unmodified (light grey) alginate gels after three days. Error bars represent the standard deviation of three biological samples. Statistical significance was independently evaluated for each RGD-modified alginate condition relative to the corresponding unmodified alginate condition using a two-tailed *t*-test evaluated with a 95% (*) or 99% (**) confidence interval.

First, we studied the effects of modifying alginate with peptides that support cell adhesion on the migration of human U87 glioblastoma cells sandwiched between tissue culture plastic (TCPS), with elastic modulus >1 GPa, and alginate hydrogels at different concentrations (with elastic moduli ranging between 0.1 and 10 kPa) (Table 1). Similar to our previous studies of cell migration using unmodified alginate [30], we observed strong migration of cells into RGD-modified alginate (Figure 1a and Figure S1). Furthermore, the rate of cell migration was strongly dependent on RGD-alginate concentration, with the rates of cell migration increasing with the increase in alginate concentration (Figure 1b). The alginate mechanical properties (i.e., storage modulus (G’)) were positively correlated with the polymer concentration and were independent of RGD modification (Table 1). Therefore, it seems that the observed rates of cell migration into RGD-alginate are in contrast to those reported for unmodified alginate scaffolds, wherein the rates of migration decreased with the increase in alginate stiffness (Figure 1b). Similar observations were also made in experiments that used another human glioblastoma cell line, U251 (Figure S2). Taken together, these results indicate that alginate stiffness may play an important role in 2.5D cell migration, but its effects may differ in the presence and absence of cell adhesion moieties.

### 3.2. RGD Density Positively Influences Rate of 2.5D Cell Migration

**Figure 2:**
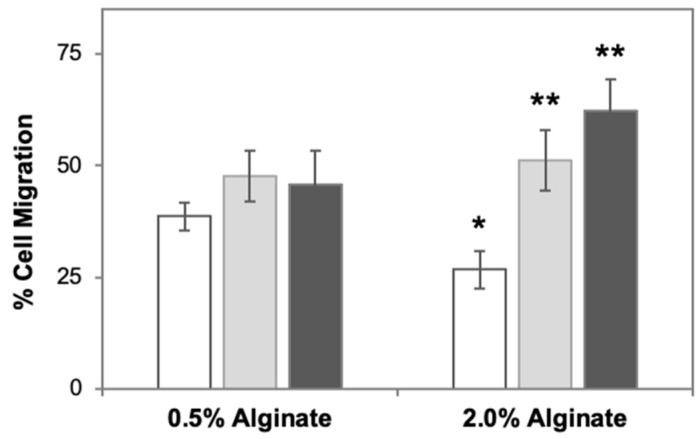
Migration of U87 cells into 0.5% and 2% alginate gels containing different ratios of RGD-modified and unmodified alginate—1:3 (white), 1:1 (light grey), and 3:1 (dark grey)—after three days. Error bars represent the standard deviation of three biological samples. Statistical significance was independently evaluated for alginate gels containing different ratios of RGD-modified and unmodified alginate relative to unmodified alginate gels using a two-tailed *t*-test evaluated with a 95% (*) or 99% (**) confidence interval.

Next, we proceeded to investigate the discrepancies between RGD-modified and unmodified alginate. Previous investigations, including our own work, have shown that the porosity of alginate hydrogels is minimally impacted by the presence of RGD ligands [25,36]. It is important to note that in the aforementioned experiments (Figure 1), both RGD density and stiffness increased with the increase in %RGD-alginate. To better understand the role of adhesion density, we prepared hybrid gels that presented varying densities of cell adhesion moieties while maintaining constant stiffness. Different ratios of RGD-modified to unmodified alginate (1:3, 1:1, and 3:1) were mixed together to yield final alginate concentrations of 0.5% and 2%, thereby enabling us to probe the effects of varying RGD densities on cell migration at the two stiffness values (Figure 2 and Figure S3). At 2% alginate, the increase in RGD resulted in increased cell migration. However, for different RGD densities at 0.5% alginate, differences in cell migration were indistinguishable, possibly due to statistically insignificant differences between cell migration with unmodified alginate and that with RGD-alginate at this concentration.

**Figure 3:**
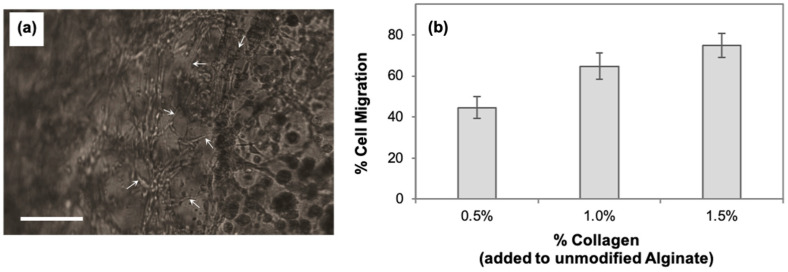
(**a**) Representative phase-contrast image showing migrated U87 cells into 0.5% unmodified alginate scaffolds containing 1% collagen on day 3. The scale bar depicts 100 µm. The image shows that cells extended processes in alginate containing collagen (as indicated by the white arrows). (**b**) Migration of U87 cells into 0.5% unmodified alginate scaffolds containing collagen in different amounts after three days. Error bars represent the standard deviation of three biological samples.

To confirm that the changes in cell migration at different densities of cell adhesion were not limited to higher stiffness (2% alginate), we adopted the approach detailed by Ulrich et al., who created hybrid gels using agarose (which does not support cell adhesion) and collagen (which supports cell adhesion) to study cell migration [37]. Collagen was an attractive choice as the second biomaterial for our studies, as it minimally changes the stiffness of alginate matrix (as verified using rheological characterization; Table S1) while providing a high density of cell adhesion moieties to support cell migration (Figure 3a). When collagen in varying amounts was added to 0.5% unmodified alginate, increased cell migration was observed at increased collagen concentrations (Figure 3b and Figure S4). Interestingly, collagen–alginate scaffolds resulted in seemingly higher rates of cell migration than RGD-alginate scaffolds, possibly due to a higher density of cell adhesion moieties in collagen–alginate scaffolds than in the RGD-alginate ones. Nevertheless, consistent with alginate and RGD-alginate matrices, increased cell migration was observed at increased concentrations of cell adhesion moieties. According to these two sets of experiments, we concluded that the increased migration rates at higher concentrations of RGD-alginate were due to increased cell attachment, not increased stiffness.

### 3.3. Reduced Role of Stiffness Gradients under Adhesion-Dependent Conditions

**Figure 4:**
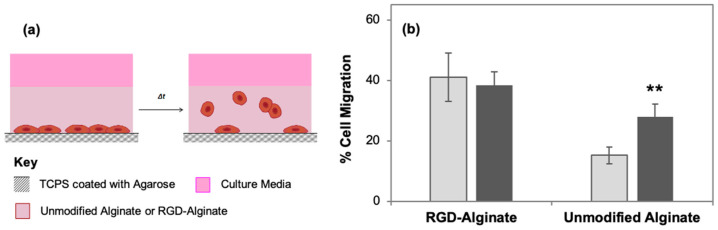
(**a**) Schematic of 2.5D cell migration platform to study the role of stiffness gradients in cell migration; the stiffness gradient can be modified by changing the stiffness of either agarose or alginate, or that of both. (**b**) Migration of U87 cells cultured on TCPS coated with either 0.5% agarose (light grey) or 3% agarose (dark grey) into 0.5% RGD-modified or unmodified alginate gels after three days. Error bars represent the standard deviation of three biological samples. Statistical significance was independently evaluated for TCPS coated with 3% agarose samples relative to TCPS coated with 0.5% agarose samples using a two-tailed *t*-test evaluated with a 95% (*) or 99% (**) confidence interval.

It is important to note that changes in alginate concentration not only change the matrix stiffness (into which the cells are migrating) but also the magnitude of the stiffness gradient (between TCPS and alginate). To better understand the role of stiffness gradients in cell migration under adhesion-dependent conditions, we performed additional experiments using TCPS coated with agarose at varying concentrations to modify its stiffness and thus the stiffness gradient (Figure 4a and Table S2). Alginate concentration was held constant at 0.5%, as this concentration resulted in similar rates of migration into RGD-modified and unmodified alginate in previous experiments. Cell migration into unmodified alginate was impacted by the changes in agarose concentration, with increased cell migration having been observed at the highest stiffness gradient, i.e., 0.5% alginate and 3% agarose-coated TCPS (Figure 4b). However, cell migration into RGD-modified alginate was not significantly impacted by changes in stiffness gradients, indicating the reduced role of stiffness gradients in adhesion-dependent migration relative to the adhesion-independent mode (Figure 4b).

### 3.4. Inhibition of Rho GTPases and Cell Adhesions Influence 2.5D Cell Migration

**Figure 5:**
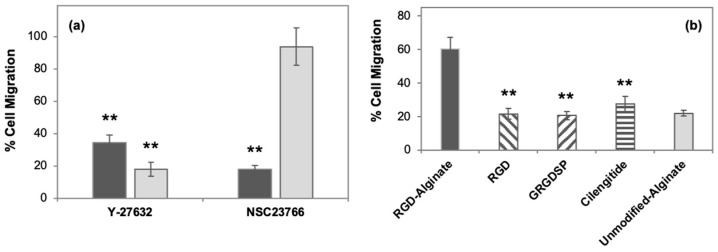
Migration of U87 cells into RGD-modified (dark grey) and unmodified (light grey) alginate gels in the presence of (**a**) Rho GTPase inhibitors Y-27632 and NSC23766, and (**b**) integrin inhibitors RGD, GRGDSP, and cilengitide relative to cell migration in the absence of inhibitors after three days. Error bars represent the standard deviation of three biological samples. Statistical significance was independently evaluated for each inhibitor condition relative to the no-inhibitor control using a two-tailed *t*-test evaluated with a 95% (*) or 99% (**) confidence interval.

Previous studies have indicated the importance of RhoA/ROCK pathways in cell migration; therefore, we sought to test the role of Rho GTPases in cell migration using small-molecule inhibitors targeting the RhoA-ROCK (Y-27632) and Rac (NSC23766) pathways. With unmodified alginate gels, inhibition was only observed in the presence of Y-27632 (Figure 5), suggesting the importance of the RhoA-ROCK-myosin II pathway (but not the Rac pathway), which is consistent with previous reports that indicate the importance of RhoA-ROCK in adhesion-independent migration [30,38]. With RGD-modified alginate gels, however, inhibition was observed in the presence of both Y-27632 and NSC23766, indicating that cell migration utilizes both the RhoA-ROCK-myosin II and Rac pathways, in contrast to independent cell migration, which only depends on the RhoA/ROCK pathway (Figure 5a). This result is not entirely surprising, as previous papers have shown that adhesion-dependent migration is a result of a biphasic reaction associated with these two pathways, such that early adhesion formation is dependent on the Rac pathway and mature focal adhesion formation is dependent on the RhoA pathway [39,40].

**Figure 6:**
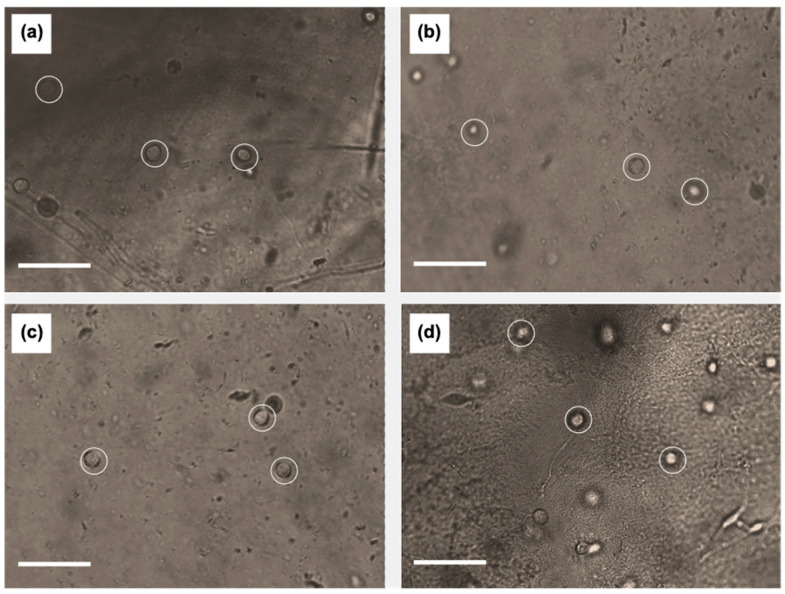
Representative pictures showing migrated U87 cells into RGD-modified alginate scaffolds in the presence of integrin inhibitors (**a**) RGD, (**b**) GRGDSP, and (**c**) cilengitide, and (**d**) unmodified alginate on day 3. The scale bar depicts 100 µm. The images show that cells (representative cells circled in white) did not extend processes in RGD-modified alginate in the presence of integrin inhibitors or in unmodified alginate.

Finally, we repeated the migration assays using commercially available inhibitors of integrin-mediated adhesions (RGD, GRGDSP, and cilengitide) as a further confirmation of the significance of focal adhesions in cell migration (Figure 5b). The presence of integrin inhibitors significantly reduced the migration rates for RGD-alginate, but it did not completely prevent cell migration. Furthermore, the rates of cell migration for cells in 1% RGD-alginate in the presence of integrin inhibitors were similar to those in 1% unmodified alginate. (Please note that cell migration in unmodified alginate was not impacted in the presence of RGD inhibitors, which is consistent with our previous study [30]). Furthermore, the images of cells in RGD-alginate in the presence of integrin inhibitors and unmodified alginate were similar and did not exhibit any cellular extensions (Figure 6). To confirm that the migrated cells were rounded under the aforementioned conditions due to the lack of cellular extensions and not due to compromised cell viability, we tested if the presence of integrin inhibitors led to differences in the viability of cells in RGD-alginate. We did not observe any significant toxicity due to integrin inhibitors; the viability of the cells migrated into alginate was greater than 80% under all the tested conditions (Figure S5). Together, these results indicate that cells may switch from adhesion-dependent migration to independent migration in the presence of integrin inhibitors.

## 4. Discussion

Metastasis is the most frequent cause of cancer-related death, and there is a critical need for more in vitro models that better capture the complexities of in vivo cell migration to expand on the existing portfolio for cancer treatment [11,41]. In order to migrate and invade tissues, cancer cells must modify their shape and stiffness, which enables them to interact with the ECM [42]. This involves cells disassembling, reorganizing, or assembling their actin cytoskeleton through time and space to generate forward movement. Upon reorganizing their cytoskeleton, cells are able to initiate cell migration by becoming polarized and elongating, and this creates a leading edge of the cell. Once the leading edge reaches the ECM, it attaches to it by binding to adhesion molecules, most commonly integrin receptors. The integrin receptors then cluster together to form a focal adhesion, the assembly of which is dynamic and changes as the cell moves. A large variety of signaling molecules are involved in this process, but the Rho GTPase family appears to be essential for the promotion of integrin-based matrix adhesion complexes. In vivo, the overexpression of Rho GTPase and its signaling components is a requirement for breast cancer metastasis, as Rho GTPase promotes cell-cycle progression by reducing cellular adhesion and promoting migration [43].

With this guidance of in vivo studies, several in vitro mammalian cell culture platforms have been developed to investigate cancer cell migration. Two-dimensional culture platforms have been utilized to probe complex biological phenomena, such as stem cell differentiation and cell migration [44,45,46,47,48]. They have also been used to understand the dynamic relationships between cells and the tumor microenvironment [49,50]. Although much has been discovered by modeling cell migration on 2D surfaces, 2D cell migration does not fully model the complexity of the migration of cells through the 3D ECM. Cell migration in 2D space is governed by adhesion and tractile forces, but other factors experienced by cells in a 3D microenvironment, including biochemical, steric, and mechanical factors, are implicated in in vivo migration [28,51,52]. For example, using a 3D fibronectin–matrigel platform, Zaman et al. demonstrated that the migration speed in 3D space is jointly influenced by two key variables: matrix density (which includes sterics and stiffness) and ligand density [28]. Three-dimensional scaffolds have also been used to develop models to explain the versatility in migration behaviors of tumor cells in complex environments, for instance, their ability to switch between mesenchymal- and amoeboid-type migration [52]. Three-dimensional platforms have also been developed to better understand the effects that local changes in stiffness have on cell migration by employing collagen scaffolds with 3D stiffness gradients [53].

While current 3D models bring in vitro platforms closer to mimicking in vivo cell environments, they still do not fully capture the heterogenous tumor microenvironment that cancer cells experience. Most platforms are homogenous and do not account for the variability that migrating cells encounter [3], such as changes in local extracellular matrix stiffness. There have been limited studies where 3D platforms have been developed to probe the effects that local changes in stiffness have on cell migration, such as a study that employed collagen scaffolds with 3D stiffness gradients [53]. Therefore, we elected to be more deliberate in studying stiffness gradients, ligand densities, and the relationship between these two factors. In a previous effort, we reported the use of a sandwich culture platform in which cells were placed between stiff TCPS and soft alginate to probe the role of stiffness gradients in adhesion-independent cell migration [30]. In the current study, we extended the utility of the platform to study adhesion-dependent migration using RGD-modified alginate, or alginate–collagen hybrid hydrogels that support integrin-based focal adhesions. As before, a range of stiffness gradients was achieved by changing the stiffness of the overlying alginate matrix (by changing the concentration of alginate) and by coating TCPS with agarose at varying concentrations. This modified platform made it possible to study the relationship between stiffness gradients and the density of adhesion ligands in cell migration. Specifically, we found that stiffness gradients played an important role in cell migration; however, their effect differed in the presence and absence of adhesion moieties. Furthermore, we observed increased cell migration in scaffolds presenting increased density of adhesion moieties, and the increased migration was not dependent on stiffness gradients, which was confirmed using both RGD and collagen-modified alginate. Finally, we tested the effects of Rho GTPase and integrin inhibitors and determined that cells can switch from adhesion-dependent migration to independent migration in the presence of integrin inhibitors, i.e., under conditions that prevent cell attachment to the matrix.

## 5. Conclusions

Cell migration is essential to many biological phenomena, such as disease progression and cancer metastasis. Current in vitro culture models do not fully capture the effects that stiffness gradients have on cell migration, yet cells experience stiffness gradients in vivo. Collectively, the results reported here capture the effects of stiffness gradients and density of adhesion ligands on cell migration and investigate their individual and combined effects. Specifically, we found that stiffness gradients play an important role in cell migration with a gradient, but the effects may differ for adhesion-dependent and -independent migration. The results reported herein indicate that adhesion-dependent migration is more impacted by cell adhesion ligand densities than by stiffness gradients. Additionally, by using commercially available inhibitors, we demonstrated the ability of cells to switch migration modes from adhesion-dependent migration to independent migration. We believe that the model system described here, which examines the effects of both biochemical and mechanical stimuli on cell migration, could facilitate the deliberate and rational design of in vitro culture platforms that better mimic the complexities of in vivo cancer cell migration.

## Figures and Tables

**Table 1:** Modulus of alginate hydrogels characterized using rotational rheology.

Alginate Concentration (*w*/*v*)	Storage Modulus (Pa)	
	RGD-Alginate	Unmodified Alginate
0.5%	502 ± 63	544 ± 43
1%	2832 ± 348	3045 ± 338
2%	7128 ± 684	6882 ± 773

## Data Availability

Data will be made available upon request by the corresponding author.

## Supplementary Materials

The following supporting information can be downloaded at: https://www.mdpi.com/article/10.3390/cancers15061729/s1, Figure S1: Qualitative microscopic analysis to track and observe migration of cells into alginate, Figure S2: Migration of U251 cells into 0.5%, 1%, and 2% RGD-modified and unmodified alginate gels after three days, Figure S3: Migration of U251 cells into 0.5% and 2% alginate gels containing different ratios of RGD-modified and unmodified alginate after three days, Figure S4: Migration of U251 cells into 0.5% unmodified alginate scaffolds containing collagen in different amounts after three days, Figure S5: Cell viability in alginate gels in the presence of integrin inhibitors, Table S1: Modulus of collagen–alginate hydrogels characterized using rotational rheology, Table S2: Modulus of agarose hydrogels characterized using rotational rheology.
