# Supplementary material for: Sandwich Culture Platforms to Investigate the Roles of Stiffness Gradients and Cell–Matrix Adhesions in Cancer Cell Migration
Source: Cancers (Basel). 2023 Mar 12;15(6):1729. doi: 10.3390/cancers15061729 (PMC10046033; doi:10.3390/cancers15061729)
Supplement: Supplementary file 1 [file cancers-15-01729-s001.zip › cancers-2179548-supplementary.pdf]

Supplementary Materials

Sandwich culture platforms to investigate the role of stiffness gradients and cell-matrix adhesions on cancer cell migration

Evangelia Bouzos and Prashanth Asuri*

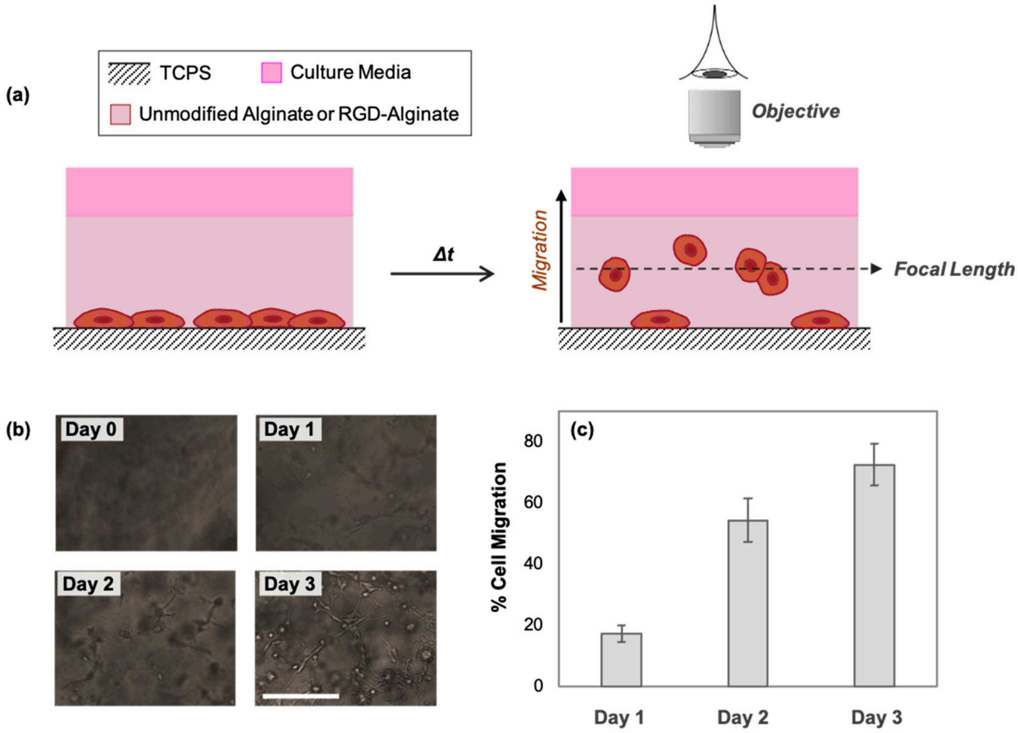

Figure S1. (a) Schematic of the qualitative microscopic analysis to track and observe migration of cells into alginate; pictures of cell migration were taken at a specific focal height at various time points. Note that the line denoting the focal height is not to scale and is for representative purposes only. (b) Representative phase contrast images showing U87 cells that migrated into alginate scaffolds (taken at a specific focal height) on days 1-3. The scale bar depicts 100 μm. (c) Migration of U87 cells into 2% RGD-alginate gels on days 1-3, as quantified using the WST assay. Error bars represent the standard deviation of three biological samples.

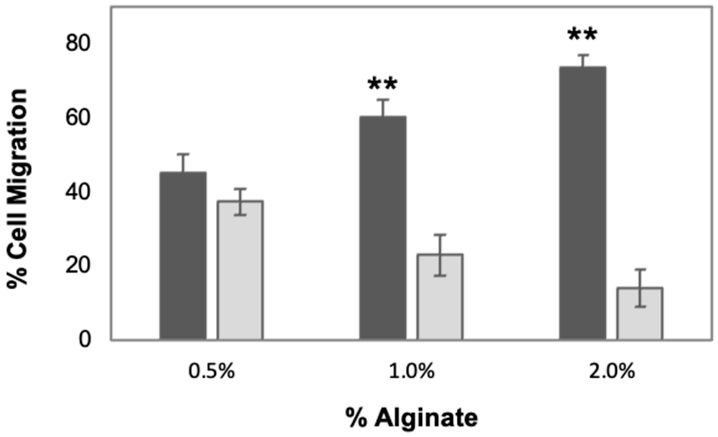

Figure S2. Migration of U251 cells into 0.5%, 1%, and 2% RGD-modified (dark grey) and unmodified (light grey) alginate gels after three days. Error bars represent the standard deviation of three biological samples. Statistical significance was independently evaluated for each RGD-modified alginate condition relative to the corresponding unmodified alginate condition using a two-tailed t-test evaluated at the 95% (*) or 99% (**) confidence interval.

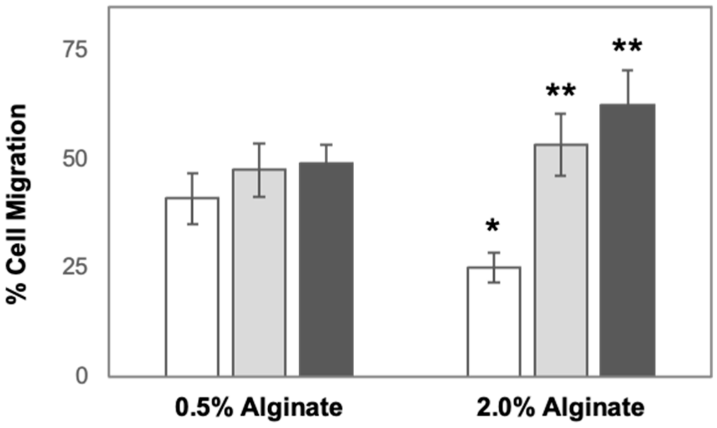

Figure S3. Migration of U251 cells into 0.5% and 2% alginate gels containing different ratios of RGD-modified and unmodified alginate – 1:3 (white), 1:1 (light grey), and 3:1 (dark grey) after three days. Error bars represent the standard deviation of three biological samples. Statistical significance was independently evaluated for alginate gels containing different ratios of RGD-modified and unmodified alginate relative to unmodified alginate gels using a two-tailed t-test evaluated at the 95% (*) or 99% (**) confidence interval.

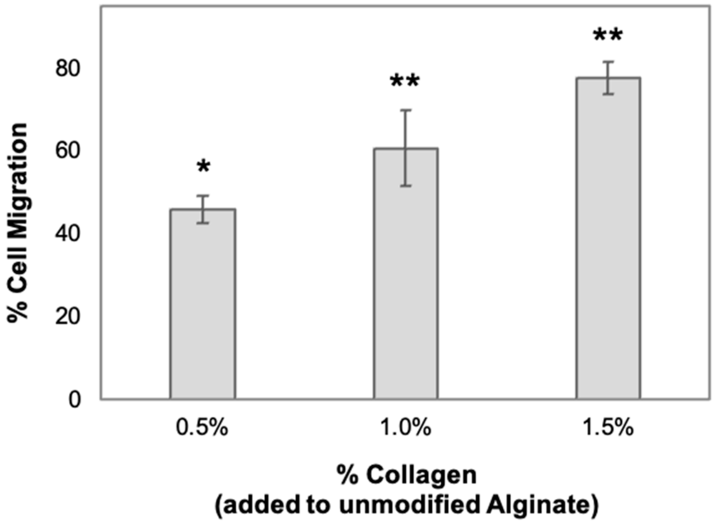

Figure S4. Migration of U251 cells into 0.5% unmodified alginate scaffolds containing different amounts of collagen after three days. Error bars represent the standard deviation of three biological samples. Statistical significance was independently evaluated for the collagen-alginate hybrid gels relative to un-modified alginate gels using a two-tailed t-test evaluated at the 95% (*) or 99% (**) confidence interval.

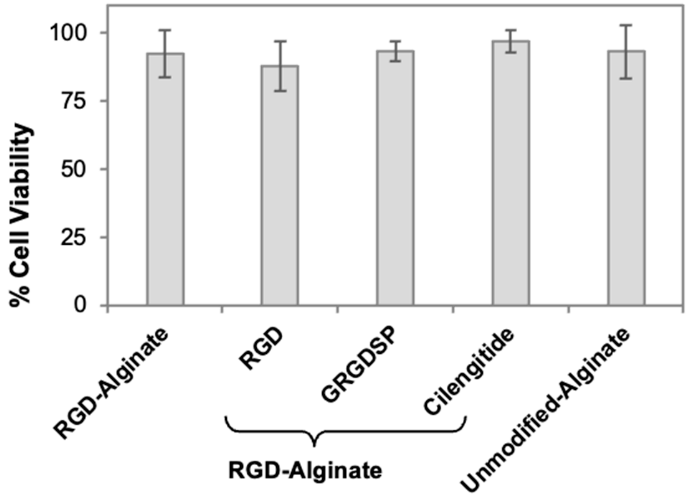

Figure S5. Percent cell viability of U87 cells migrated into RGD-modified alginate gels, RGD-modified alginate gels in the presence of RGD, GRGDSP, and cilengitide, and unmodified alginate gels after three days, as determined by trypan blue exclusion assay*. Error bars represent the standard deviation of three biological samples.

**Trypan blue exclusion assay: U87 cells in the alginate digest (for all the tested conditions) were centrifuged, resuspended in serum-free DMEM containing trypan blue, incubated for ca. 3 minutes, and then examined to determine the percentage of cells that have clear cytoplasm (viable cells) versus cells that have blue cytoplasm (nonviable cells) using a hemacytometer.*

Table S1. Modulus of collagen-alginate hydrogels as characterized by rotational rheology.

Alginate Concentration (w/v)	Collagen Concentration (w/v)	Storage Modulus (Pa)
0.5%	0.5%	514 ± 81
0.5%	1%	532 ± 42
0.5%	1.5%	528 ± 71

Table S2. Modulus of agarose hydrogels as characterized by rotational rheology.

Agarose Concentration (w/v)	Storage Modulus (Pa)
0.5%	824 ± 59
3%	11,453 ± 1278
